# New Function of the Adaptor Protein SH2B1 in Brain-Derived Neurotrophic Factor-Induced Neurite Outgrowth

**DOI:** 10.1371/journal.pone.0079619

**Published:** 2013-11-15

**Authors:** Chien-Hung Shih, Chien-Jen Chen, Linyi Chen

**Affiliations:** 1 Institute of Molecular Medicine, National Tsing Hua University, Hsinchu, Taiwan, Republic of China; 2 Department of Medical Science, National Tsing Hua University, Hsinchu, Taiwan, Republic of China; Hungarian Academy of Sciences, Hungary

## Abstract

Neurite outgrowth is an essential process for the establishment of the nervous system. Brain-derived neurotrophic factor (BDNF) binds to its receptor TrkB and regulates axonal and dendritic morphology of neurons through signal transduction and gene expression. SH2B1 is a signaling adaptor protein that regulates cellular signaling in various physiological processes. The purpose of this study is to investigate the role of SH2B1 in the development of the central nervous system. In this study, we show that knocking down SH2B1 reduces neurite formation of cortical neurons whereas overexpression of SH2B1β promotes the development of hippocampal neurons. We further demonstrate that SH2B1β promotes BDNF-induced neurite outgrowth and signaling using the established PC12 cells stably expressing TrkB, SH2B1β or SH2B1β mutants. Our data indicate that overexpressing SH2B1β enhances BDNF-induced MEK-ERK1/2, and PI3K-AKT signaling pathways. Inhibition of MEK-ERK1/2 and PI3K-AKT pathways by specific inhibitors suggest that these two pathways are required for SH2B1β-promoted BDNF-induced neurite outgrowth. Moreover, SH2B1β enhances BDNF-stimulated phosphorylation of signal transducer and activator of transcription 3 at serine 727. Finally, our data indicate that the SH2 domain and tyrosine phosphorylation of SH2B1β contribute to BDNF-induced signaling pathways and neurite outgrowth. Taken together, these findings demonstrate that SH2B1β promotes BDNF-induced neurite outgrowth through enhancing pathways involved MEK-ERK1/2 and PI3K-AKT.

## Introduction

Development of the nervous system depends on both extracellular cues and intrinsic factors, including neurotrophins, to promote differentiation. Neurotrophins are a family of closely related proteins that regulate many aspects of survival, development, maintenance and function of neurons in both the peripheral and the central nervous systems (PNS and CNS) [1]. Brain-derived neurotrophic factor (BDNF) is a neurotrophic factor that was originally shown to promote survival of a subpopulation of dorsal root ganglion neurons [2]. Its specific receptor TrkB is highly expressed in the developing CNS (brain and spinal cord) and PNS (cranial and spinal ganglia) [3], [4]. By binding to its receptor TrkB, BDNF acts in a paracrine and autocrine manner to control a variety of brain processes, including promoting neurite outgrowth of developing retinal ganglion cells (RGCs) [5], increasing survival and axon outgrowth from pontocerebellar mossy fiber neurons in vitro [6]. Besides axonal development, BDNF and TrkB also play important roles in regulating the growth and branching of dendrites in cortical neurons [7]–[10].

Three major intracellular signaling pathways have been implicated by BDNF-TrkB binding. There are the pathways involving Ras/Raf/Mitogen-activated protein kinase (MAPK)/ERK kinase (MEK) activation of extracellular signal-regulated kinase (ERK), phosphatidyl inositol-3-kinase (PI3K) stimulation of AKT and PLCγ1-dependent generation of inositol triphosphate and diacylglycerol, leading to mobilization of Ca^2+^ stores and activation of DAG-regulated protein kinases [1], [11]–[13]. ERK signaling pathway has been shown to be required for TrkB-mediated axonal outgrowth in sympathetic neurons and neuronal differentiation of PC12 cells ectopically expressing TrkB [14]–[16]. AKT has recently been implicated in several aspects of neurite outgrowth, including elongation, branching, calibre and survival [15], [17]–[19]. These findings suggest that MEK-ERK and PI3K-AKT signaling pathways regulate neurite elongation, branching and calibre of PC12 cells and primary neurons.

SH2B1β is a member of the SH2B family of adaptor proteins including SH2-B (SH2B1), APS (SH2B2) and Lnk (SH2B3). SH2B1 contains three proline-rich domains, a pleckstrin homology domain, a dimerization domain, and a carboxy (C)-terminal Src homology (SH2) domain. Four SH2B1 splice variants, α, β, γ, and δ, differ only in their C-termini starting just past the SH2 domain [20]. SH2B1β is known to interact with the activated forms of Janus kinase 2, nerve growth factor (NGF) receptor TrkA, platelet-derived growth factor receptor, glial cell line-derived neurotrophic factor receptor (GDNFR) and fibroblast growth factor receptor 3 (FGFR3) through its SH2 domain and then is tyrosyl phosphorylated by these receptors to mediate downstream signaling pathways [21]–[28]. Previous studies also revealed a positive role of SH2B1β in NGF-, GDNF- and FGF1-induced neurite outgrowth of dorsal root ganglion and PC12 cells [21], [24], [26], [29], model systems for the peripheral nervous system. The cellular role of SH2B1β, the predominant splice variant in the nervous system, in the CNS has not been investigated. In this study, we examine whether SH2B1β regulates neurite development of cortical and hippocampal neurons. As BDNF is a predominant neurotrophin in the central nervous system, we further determine whether SH2B1β is involved in BDNF signaling during neuronal differentiation.

## Materials and Methods

### Animal handling. Ethics statement

All experiments were conducted in accordance with the guidelines of the Laboratory Animal Center of National Tsing Hua University (NTHU). Animal use protocols were reviewed and approved by the NTHU Institutional Animal Care and Use Committee (Approval number 09837)∼

### Antibodies and reagents

Polyclonal antibody to rat SH2B1β was raised against a glutathione S-transferase fusion protein containing amino acids 527–670 of SH2B1β as described previously [23]. Rat tail collagen I was purchased from BD Bioscience (Franklin Lakes, NJ). Human brain-derived neurotrophic factor was purchased from PeproTech (Rocky Hill, NJ). Protein G agarose beads and rabbit anti-pTrkB(Y706) were purchased from Santa Cruz Biotechnology (Santa Cruz, CA). Mouse monoclonal antibody against phospho-Tyrosine (4G10) was purchased from Millopore (Billerica, MA). Lipofectamine 2000, Alexa Flour 700 goat anti-mouse IgG secondary antibody, powder of Dulbecco’s Modified Eagle Medium (DMEM), horse serum, fetal bovine serum, L-glutamine, antibiotic-antimycotic, G418 and Zeocin were purchased from Invitrogen (Carlsbad, CA). IRDye800CW-labeled anti-rabbit secondary antibody was from LI-COR Biosciences (Lincoln, NE). Mouse anti-myc tag antibody was purchased from Hopegen Biotechnology Development Enterprise (Taipei, Taiwan) Anti-ERK1/2 antibody was purchased from Sigma-Aldrich (St. Louis, MO). Anti-AKT, anti-STAT3, anti-pERK1/2, anti-pAKT(S473), anti-PLCγ and anti-pPLCγ(Y783) were purchased from Cell Signaling (Danvers, MA). Anti-pSTAT3(S727) antibody was purchased from Bioworld Technology (Minneapolis, MN). Anti-TrkB was purchased from BD Transduction Laboratories. LY294002 and U0126 were from Calbiochem (San Diego, CA). BCA assay reagent was purchased from Santa Cruz Biotechnology (Santa Cruz, CA).

### Primary culture of cortical and hippocampal neurons

Brain cortex and hippocampus were dissected from embryonic day 18 (E18) embryos of Sprague-Dawley rats (purchased from BioLASCO Taiwan Co., Ltd.), and treated with papain (10 U/ml) to dissociate cells. Dissociated cells were washed and re-suspended in minimal essential medium (MEM) supplemented with 5% HS and 5% FBS. Neurons were then plated onto 30 µg/ml poly-L-lysine-coated coverslips or dishes and cultured in neurobasal medium with B27 (containing additional 0.025 mM glutamate) on DIV (day *in vitro*) 1. On DIV 3, cells were treated with 5 µM cytosine 1-β-D-arabinofuranoside to inhibit the growth of glial cells, half of the neurobasal and glutamine medium was replaced by fresh medium every 3 day. Lipofectamine 2000 or calcium phosphate reagents were used to transfect primary neurons according to the manufacture’s instruction. 1.5-3 hours after transfection, culture medium was replaced with fresh medium.

### Knockdown of endogenous SH2B1

pLKO.1 lentiviral vector that contains oligonucleotides targeting specific gene sequence, pLKO.1-shSH2B1 (Clone ID TRCN0000247808, 0000247810, 0000247809 ) and pLKO.1-shLacZ (Clone ID TRCN0000072236, 0000231717 ) were purchased from National RNAi Core Facility, located at the Institute of Molecular Biology/Genomic Research Center, Academic Sinica, Taiwan. pLKO.1-shLacZ or pLKO.1-shSH2B1 was transiently transfected to PC12-TrkB cells or primary neurons by lipofectamine 2000 or calcium phosphate reagents. 24 hours after transfection, PC12 cells were then subjected to puromycin (5 µg/ml) selection for at least 1 month to make stable cell lines (PC12-TrkB+shLacZ and PC12-TrkB+shSH2B1).

### DNA constructs

pEGFP-C1, GFP-SH2B1β, GFP-SH2B1βR555Eβ, GFP-SH2B1β9YFβ and myc-SH2B1β were generous gifts from Dr. Christin Carter-Su at University of Michigan, USA. pBSTR1-TrkB was kindly provided by Dr. Jin-Chung Chen at Chang Gung University, Taiwan. NotI-BamHI digestion of pBSTR1-TrkB releases 3.1 kb rat TrkB cDNA. TrkB cDNA was subcloned into pCMVtag4C or pZeoSV2+ vector via NotI-BamHI sites. pCMVtag4C and pZeoSV2+ contain selectable marker providing resistance to kanamycin and Zeocin, respectively.

### Culture of PC12 cells

PC12 cells were purchased from American Type Culture Collection. PC12 cells were maintained on the collagen-coated plates (coated with 0.1 mg/ml rat-tail collagen type I in 0.02N acetic acid) and grown at 37°C in 10% CO_2_ in DMEM containing 10% horse serum (HS), 5% fetal bovine serum (FBS), 1% L-glutamine (L-Gln) and 1% antibiotic-antimycotic (AA). PC12 cells stably overexpressing TrkB/pCMVtag4C were made by transfecting TrkB to PC12 and selecting with medium containing 5 mg/ml G418. PC12 cells stably overexpressing GFP (PC12-GFP cells), GFP-SH2B1β (PC12-SH2B1β cells), GFP-SH2B1β(R555E) (PC12-R555E cells) and GFP-SH2B1β(9YF) (PC12-9YF cells) were made according to Chen et al [30]. PC12-GFP, PC12-SH2B1β, PC12-R555E and PC12-9YF cells stably overexpressing TrkB/pZeoSV2+ (PC12-GFP+TrkB, PC12-SH2B1β+TrkB, PC12-R555E+TrkB and PC12-9YF+TrkB cells) were made by transfecting TrkB to PC12-GFP, PC12-SH2B1β, PC12-R555E and PC12-9YF cells and selecting with medium containing 5 mg/ml G418 and 600 µg/ml Zeocin for at least 60 days. Pooled populations of stable clones were used to avoid clonal variation.

### Neurite outgrowth

For BDNF-induced neurite outgrowth, all stable cell lines were plated on 3.5-cm culture dishes coated with 0.1 mg/ml of collagen type I. For neuronal differentiation, PC12+TrkB cells were treated with 100 ng/ml BDNF in low-serum differentiation medium (DMEM containing 2% horse serum, 1% fetal bovine serum, 1% antibiotic-antimycotic and 1% L-glutamine). Cells were pre-treated 1 h with or without 20 µM U0126 or LY294002 before the addition of 50 ng/ml BDNF. Neurite outgrowth was monitored for 1 or 3 days. Medium containing BDNF and the inhibitor was replaced every two days. Images of differentiating cells were taken using Zeiss Observer Z1 microscope using either 10X (NA/0.3), 20X (NA/0.4) or 40X (NA/0.6) objective. The definition of morphological differentiation in PC12 cells is that the length of the neurite should be at least twice of the diameter of the cell body. The percentage of differentiation was scored as the percentage of differentiated cells per counted cells.

### Measurement of neurite length and neurite branching

The average length of the longest neurite (i.e. neurite extending directly from the cell body) was measured using the Simple Neurite Tracer, a plugin for ImageJ software to make semi-automatic tracing of neurons or tube-like structures (neurite or filopodia). The mean pixel value of neurite length was measured and converted pixels into micrometers (µm). The number of end-points were quantified using the built-in cell-counter in ImageJ software. The end point be defined as the location at the tip of filopodia and neurites.

### Immunoblotting and immunoprecipitation

Cells were treated as indicated and lysates were collected in RIPA buffer (50 mM Tris-HCl, pH 7.5, 150 mM NaCl, 2 mM EGTA, 1% Triton X-100) containing protease inhibitors (1 mM Na_3_VO_4_, 10 ng/ml leupeptin and 10 ng/ml aprotinin, 1 mM phenylmethylsulfonyl fluoride, PMSF). Protein concentration of each sample was determined by BCA protein assay. Equal amount of proteins were loaded to and resolved by sodium dodecyl sulfate-polyacrylamide gel electrophoresis (SDS-PAGE) and then transferred to nitrocellulose membrane for western blotting analysis using the indicated antibodies. The immunoblots were detected using IRDye-conjugated IgG and the Odyssey Infrared Imaging System (LI-COR Biosciences, Lincoln, NE). For assessing the phosphorylation of signaling proteins, phospho-antibodies were used to detect phosphorylated proteins before re-probing with antibodies against these signaling proteins. For immunoprecipitation, cell lysates were collected in RIPA buffer containing 1 mM Na_3_VO_4_, 1 mM PMSF, 10 ng/ml aprotinin and 10 ng/ml leupeptin and incubated with anti-myc antibody (1:200) overnight at 4°C followed by protein G agarose beads pull down. The immunoprecipitated proteins were analyzed by western blotting.

### Inhibitory assay

For inhibitor assays, PC12-GFP+TrkB and PC12-SH2B1β+TrkB cells were seeded about 80% confluency on 3.5 cm dishes. PC12-GFP+TrkB and PC12-SH2B1β+TrkB cells were grown in serum-free medium containing 1% BSA for overnight and then pre-treated with or without the indicated inhibitor 1 h before adding 50 ng/ml BDNF. At the indicated time points, cells were collected and lysed. The lysate was then analyzed via Western blotting.

### Statistical analysis

Neuronal differentiation results were expressed as mean ± standard error. Immunoblotting quantification result was expressed as mean ± S.E.M. or mean ± standard deviation (S.D.) (for Figure S1), which indicates the range of data from two different experiments. Statistical significance was determined by paired Student’s t-test. A p-value <0.05 was considered as statistically significant.

## Results

### SH2B1 regulates neurite outgrowth of cortical and hippocampal neurons

**Figure 1:**
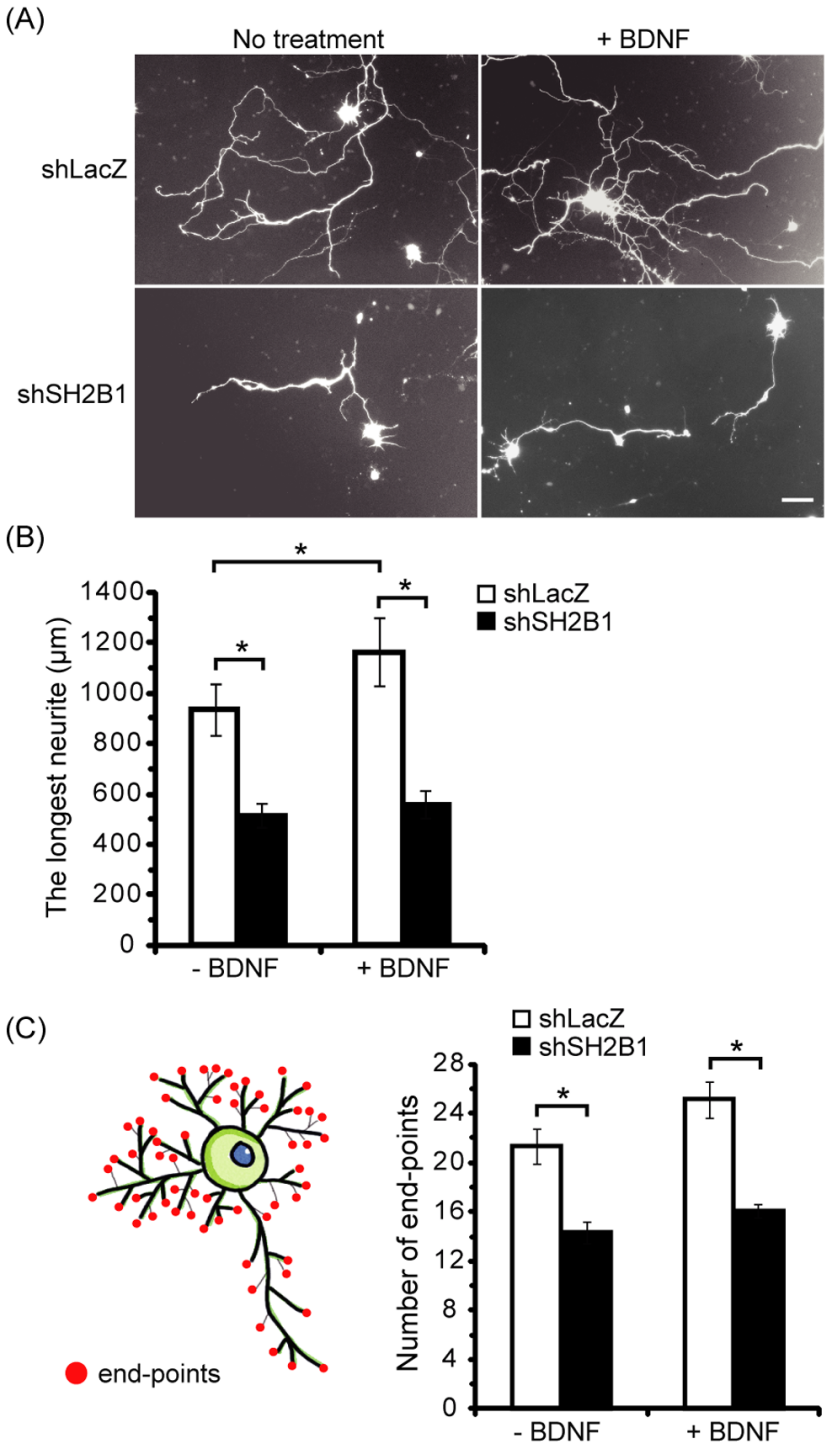
Reduction of SH2B1 expression attenuated BDNF-induced neurite outgrowth of cortical neurons. Cultured cortical neurons on DIV 4 were transfected with the shLacZ or shSH2B1 together with pEGFP vector. Twenty-four hours after transfection, cells were either untreated or treated with 50 ng/ml BDNF for 1 day. (A) Representative live cell images of primary cortical neurons are shown using Zeiss Observer Z1 microscope using 10X (NA/0.3). Scale bar  =  50 µm. (B) Average length of the longest neurite per neuron. 20–35 GFP-transfected cortical neurons were counted per condition for each experiment. Values were means ± S.E.M from three independent experiments (*: p<0.05, paired Student’s t-test). (C) Average number of end-points per neuron. 10–15 GFP-transfected cortical neurons were counted per condition for each experiment. Values were means ± S.E.M from three independent experiments (*: p<0.05, paired Student’s t-test). The length and branches of neurites were measured using ImageJ software as described in the Materials and Methods section.

**Figure 2:**
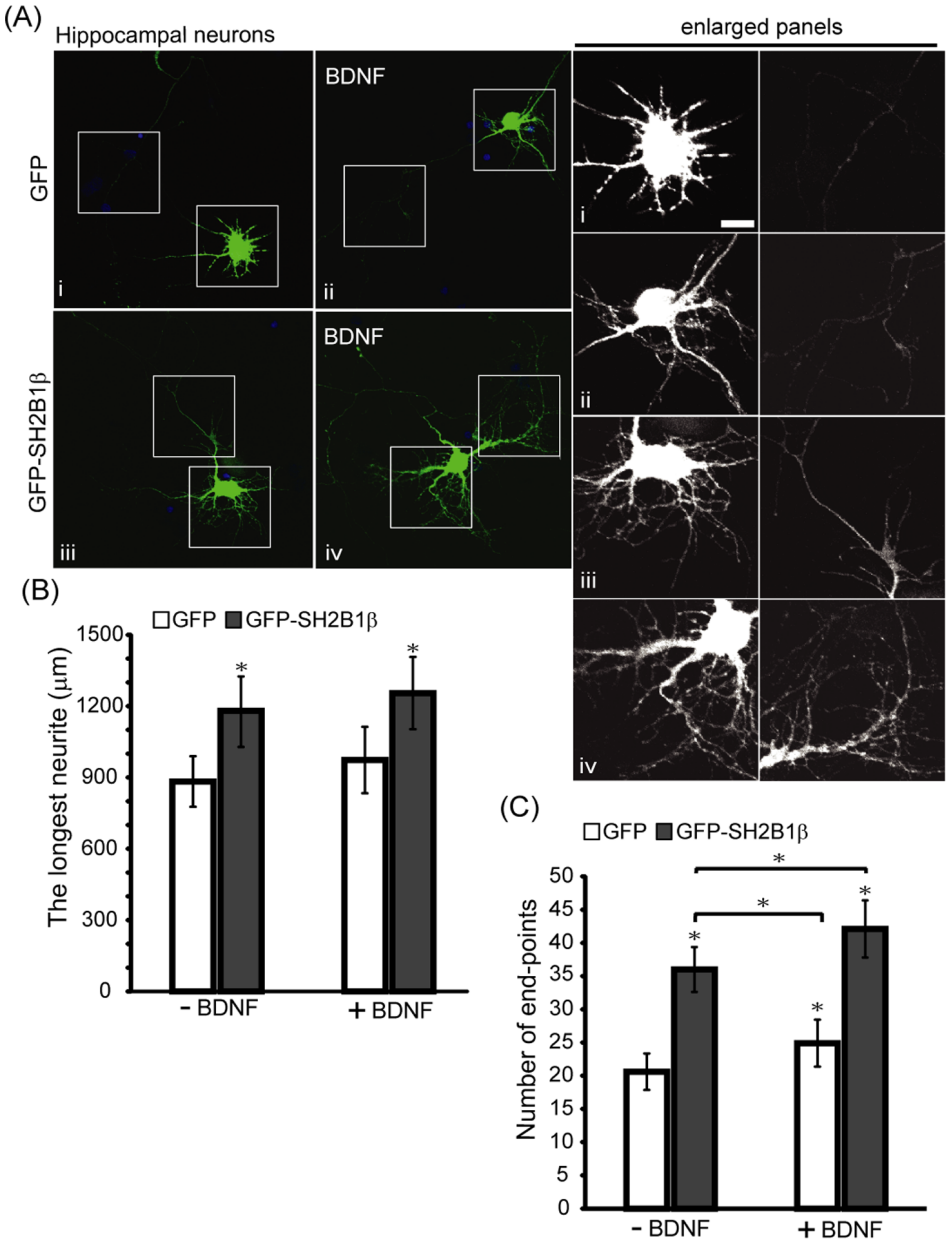
Overexpressing SH2B1β enhances BDNF-induced neurite outgrowth and branching in hippocampal neurons. (A) E18 primary hippocampal neurons were transiently transfected with either GFP or GFP-SH2B1β on DIV 4. One day after transfection, neurons were treated with 50 ng/ml BDNF for 2 days. The morphology of the neurons was visualized on DIV 7 by Zeiss LSM510 meta confocal microscope using 20X (NA/0.75) objective. Boxes mark the neurites of hippocampal neurons. Enlarged images of the neurites and branching are shown on the right panels. Scale bar  =  10 µm. (B) Neurite length of the transfected neurons were measured as described in the Materials and Methods. Average length of the longest neurite per neuron. The length of neurites were measured using Simple Neurite Tracer plug-in of the ImageJ software. (*: P<0.05, compared with GFP without BDNF). (C) The number of end-points were quantified by ImageJ software. 15–20 GFP- or GFP-SH2B1β-transfected hippocampal neurons were counted from three independent experiments. Values are mean ± SE from three independent experiments and statistically compared by student’s *t*-test (*: P<0.05).

To investigate whether SH2B1β is involved in the development of CNS, we examine the role of SH2B1β in the neurite outgrowth of embryonic day 18 (E18) cortical neurons by knocking down endogenous SH2B1. Neurite outgrowth of cortical neurons transfected with short hairpin (sh) shLacZ control plasmid was compared to those transfected with shSH2B1 construct together with pEGFP plasmid on day in vitro (DIV) 4, followed by with or without BDNF stimulation. As shown in Figure 1A, knocking down SH2B1 significantly reduced BDNF-induced neurite length and branching of cortical neurons. The quantified results are shown in Figure 1B and C. In the absence of BDNF, the average neurite length was reduced to 45%. Similarly, knocking down SH2B1 reduced the average neurite length to around 50% in the presence of BDNF (Figure 1B). Moreover, overexpression of SH2B1β enhanced neurite length and BDNF-induced branching of hippocampal neurons (Figure 2). These data suggest that SH2B1 regulates neurite outgrowth of E18 cortical and hippocampal neurons.

### Knocking down SH2B1β reduced BDNF-induced neurite outgrowth of PC12 cells

**Figure 3:**
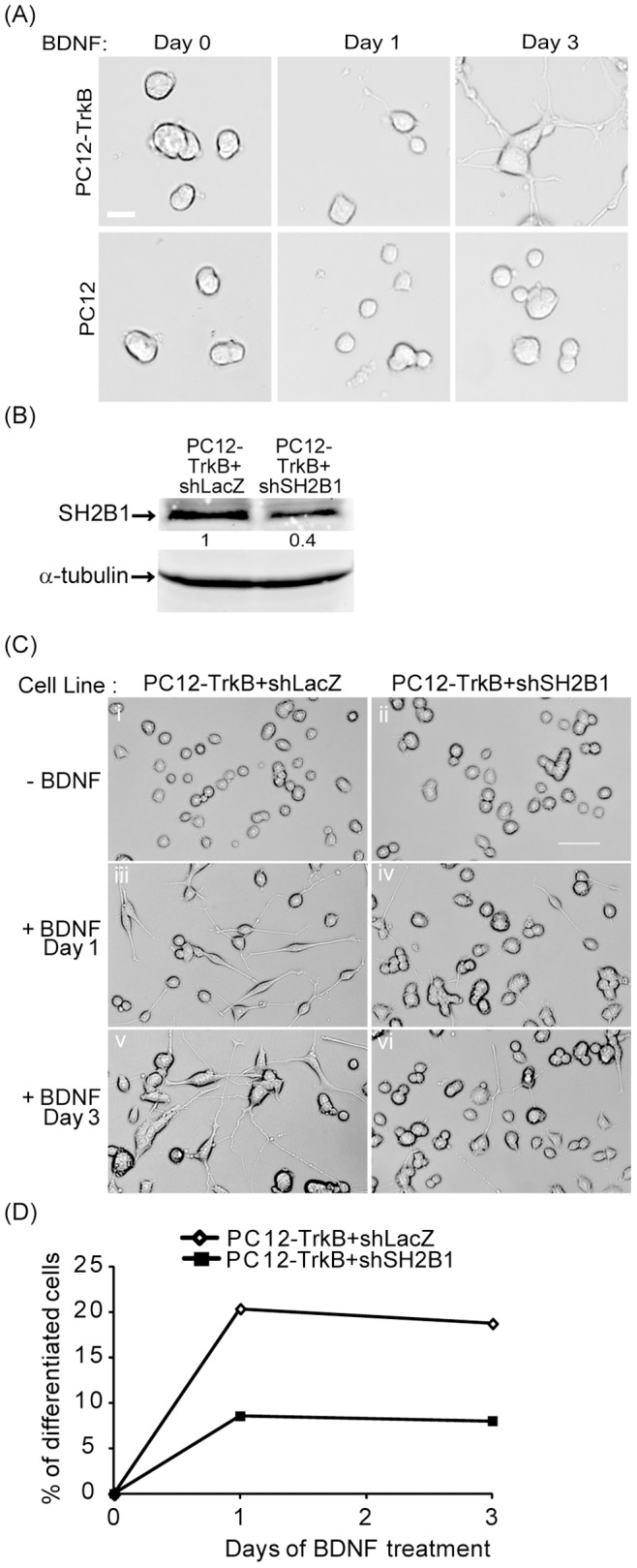
Knock-down of SH2B1 expression reduces BDNF-induced neurite outgrowth in PC12-TrkB cells. (A) PC12-TrkB stable cell line and parental PC12 cells were treated with 50 ng/ml BDNF in low serum medium for 0, 1 or 3 days. Representative live cell images are shown. Scale bar  =  20 µm. (B) PC12 cells expressing TrkB and shLacZ (PC12-TrkB+shLacZ) or TrkB and shSH2B1 (PC12-TrkB+shSH2B1) were established. Cell lysates from PC12-TrkB+shLacZ and PC12-TrkB+shSH2B1 cells were collected and analyzed via SDS-PAGE and immunoblotted with anti-SH2B1 or α-tubulin antibody. α-tubulin was used as a loading control. (C) PC12-TrkB+shLacZ cells (i, iii, v) and PC12-TrkB+shSH2B1 cells (ii, iv, vi) were treated with 100 ng/ml BDNF in low serum medium for 0 (i, ii), 1 (iii, iv) or 3 (v, vi) days. Representative live cell images are shown. Scale bar  =  50 µm. (D) The percentages of neuronal differentiation for PC12-TrkB+shLacZ and PC12-TrkB+shSH2B1 cells treated with BDNF for 1 or 3 days were quantified.

We thus set out to investigate the underlying mechanism by which SH2B1β enhances BDNF-induced neurite outgrowth. Because of the poor transfection efficiency of primary neurons, PC12 cell line was used as a model system to carry out the following experiments. PC12 cells do not express BDNF receptor, TrkB. We thus establish PC12 cell line that stably expresses TrkB (PC12-TrkB) to determine if we could re-capitulate the phenotype observed in cortical and hippocampal neurons. PC12-TrkB and PC12 cells were treated with 50 ng/ml BDNF in low serum medium for 0, 1 or 3 days. The parental PC12 cells were not responsive to BDNF and thus no neurite outgrowth was observed (Figure 3A). We compared the neurite outgrowth of PC12-TrkB cells with or without reduced endogenous SH2B1. Knocking down SH2B1 via shSH2B1 transfection, we established two other stable cell lines, PC12-TrkB+shLacZ control cell line, and PC12-TrkB+shSH2B1 cell line that expresses 60% reduced SH2B1 (Figure 3B). The definition of neuronal differentiation of PC12 cells is that the length of the neurite should be at least twice of the diameter of the cell body. Neurite outgrowth of PC12-TrkB+shSH2B1 was obviously reduced compared to control PC12-TrkB+shLacZ cells (Figure 3C). BDNF-induced neuronal differentiation was reduced by 50% when endogenous SH2B1 was reduced (Figure 3D). These results recapitulate findings using primary cortical neurons and suggest that PC12 cells expressing TrkB can be used to study how SH2B1β regulates BDNF-induced neurite outgrowth.

### Overexpressing SH2B1β enhances BDNF-induced outgrowth and signaling

**Figure 4:**
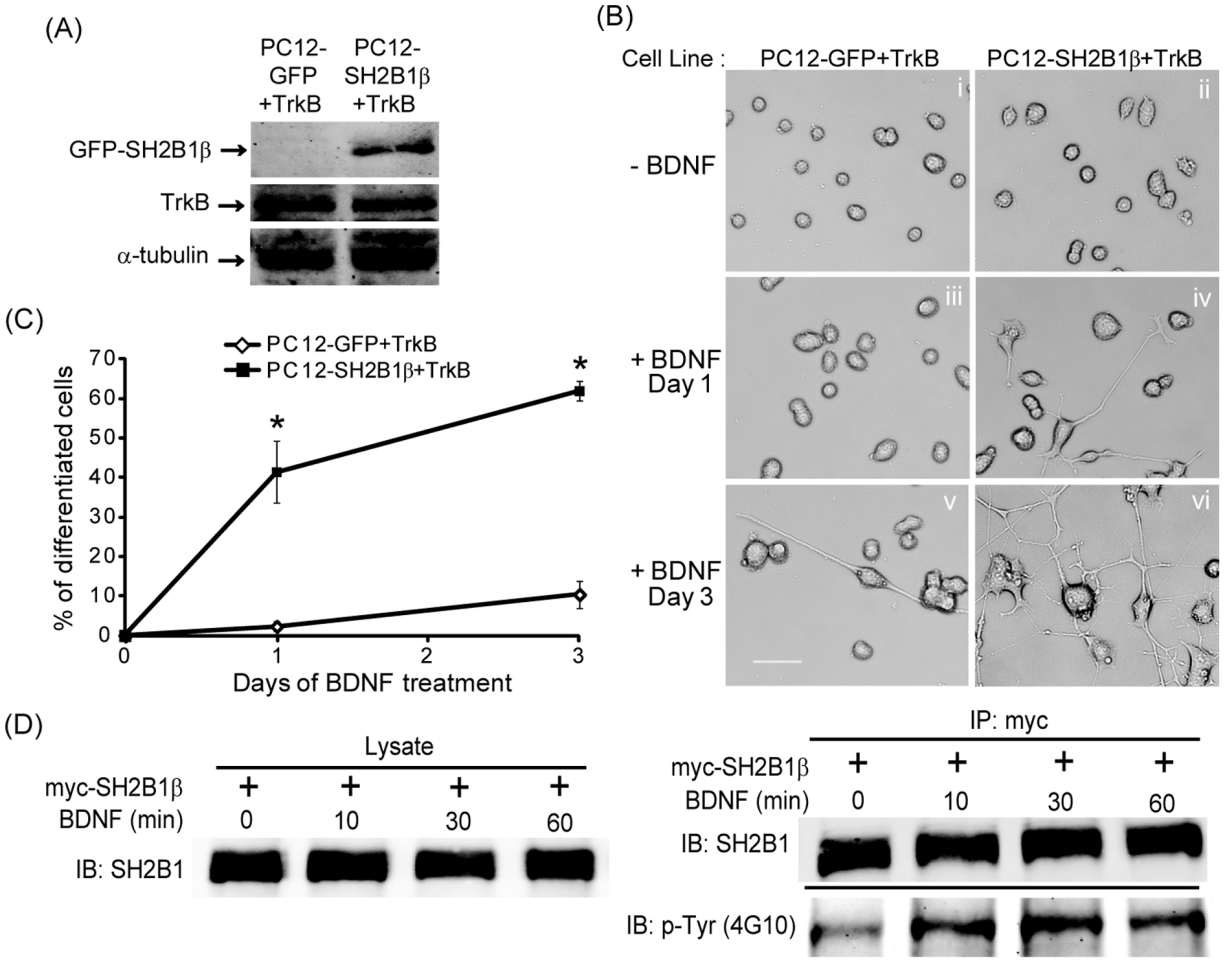
Overexpression of SH2B1β enhances BDNF-induced neurite outgrowth. PC12 cells overexpressing GFP and TrkB (PC12-GFP+TrkB) or GFP-SH2B1β and TrkB (PC12-SH2B1β+TrkB) were established. (A) Cell lysates from PC12-GFP+TrkB and PC12-SH2B1β+TrkB cells were collected and analyzed via SDS-PAGE and immunoblotted with anti-SH2B1, TrkB and α-tubulin antibodies. α-tubulin was used as a loading control. (B) PC12-GFP+TrkB cells (i, iii, v) and PC12-SH2B1β+TrkB cells (ii, iv, vi) were treated with 100 ng/ml BDNF in low serum medium for 0 (i, ii), 1 (iii, iv) or 3 (v, vi) days. Representative live cell images are shown. Scale bar  =  50 µm. (C) The percentages of neuronal differentiation for PC12-GFP+TrkB and PC12-SH2B1β+TrkB cells treated with BDNF for 1 or 3 days were quantified. Values are means ± S.E.M. from three independent experiments. (*: compared to the percentages of differentiated PC12-GFP+TrKB cells for the same BDNF treatment day, p<0.05, paired Student’s t-test). (D) E18 cortical neurons were transiently transfected with myc-SH2B1β on DIV 10. One day after transfection, neurons were treated with 50 ng/ml BDNF for 0, 10, 30, 60 mins. Cell lysates were collected and subjected to immunoprecipitation using anti-myc antibody followed by immunoblotting with anti-SH2B1 or anti-pTyr (4G10) antibody.

To understand how SH2B1β may enhance BDNF-induced signaling, two new PC12 cells stably cell lines, PC12 cells expressing GFP and TrkB receptors (PC12-GFP+TrkB) and PC12 cells stably expressing GFP-SH2B1β and TrkB receptors (PC12-SH2B1β+TrkB), were established to study the regulation of BDNF-induced neurite outgrowth by SH2B1β. Figure 4A showed the expression of GFP-SH2B1β and TrkB in these two cell lines. To examine the effects of SH2B1β on BDNF-induced neurite outgrowth, PC12-GFP+TrkB and PC12-SH2B1β+TrkB cell lines were mock-treated or treated with 100 ng/ml BDNF for 1 or 3 days. As shown in Figure 4B, PC12-SH2B1β+TrkB cells showed higher percentage of cells with neurites (Figure 4B, panels iv and vi) compared to the control PC12-GFP+TrkB cells (Figure 4B, panels iii and v) in response to BDNF treatment for 1 or 3 days. The quantified results of BDNF-induced neuronal differentiation of PC12-SH2B1β+TrkB cells were significantly higher than those of PC12-GFP+TrkB cells (Figure 4C). To determine whether SH2B1β is a signaling adaptor protein for BDNF-TrkB signaling, the BDNF-induced tyrosine phosphorylation of SH2B1β is examined. To this end, myc-SH2B1β was transfected to cortical neurons and immunoprecipitated with anti-myc antibody followed by immunoblotting with anti-pTyr antibody. In response to BDNF stimulation, tyrosine phosphorylation of SH2B1β was increased for at least 60 min (Figure 4D).

**Figure 5:**
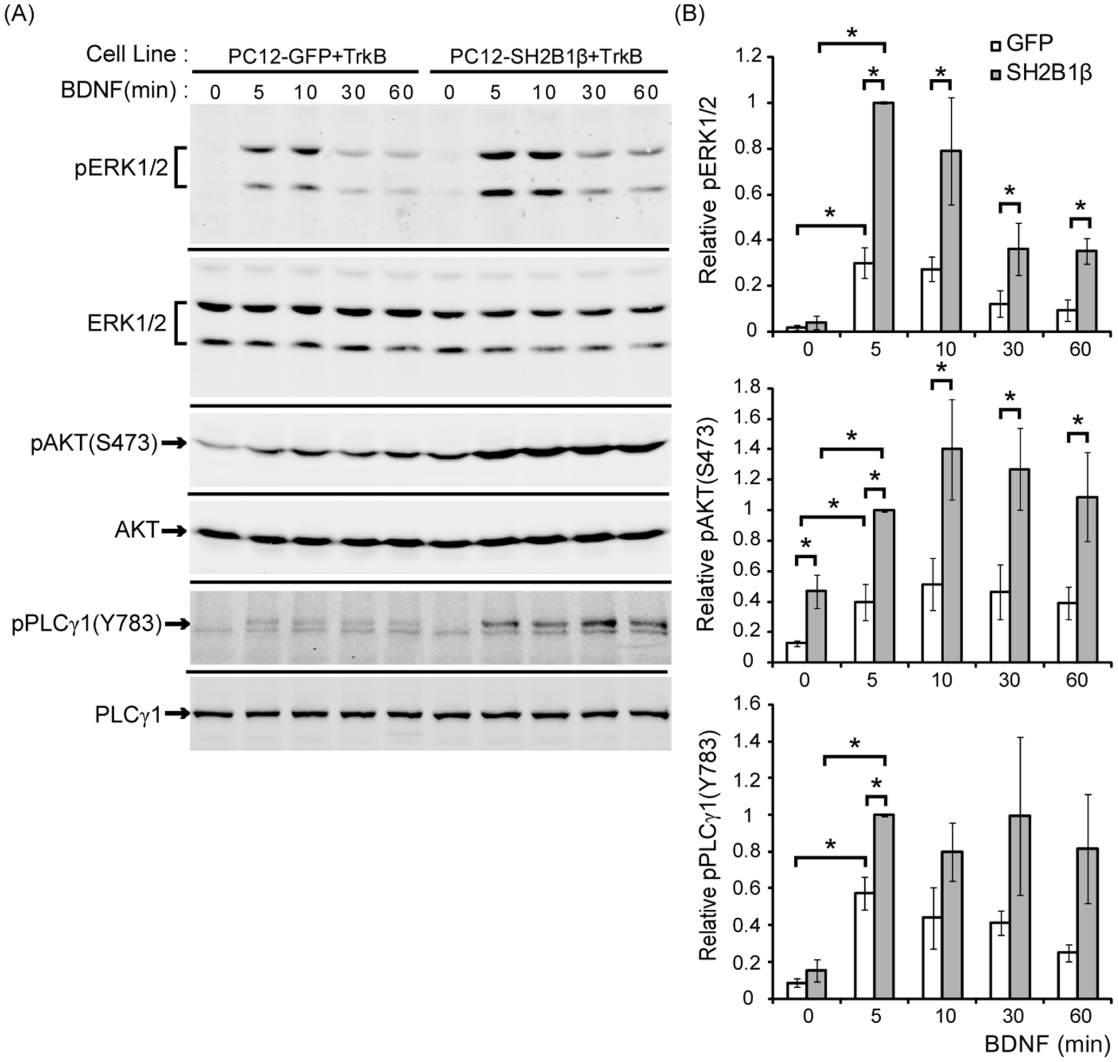
BDNF-induced phosphorylation of ERK1/2 and AKT are enhanced by overexpression of SH2B1β. (A) PC12-GFP+TrkB or PC12-SH2B1β+TrkB cells were cultured in serum-free medium overnight before stimulation of 50 ng/ml BDNF for the indicated time points. Lysates were collected and equal amount of proteins was separated by SDS-PAGE and immunoblotted with anti-pERK1/2, ERK1/2, pAKT(S473), AKT, pPLCγ1(Y783), and PLCγ1 antibodies. Representative blots from three independent experiments are shown. (B) pERK1/2, pAKT(S473), and pPLCγ1(Y783) levels were normalized to total ERK1/2, AKT and PLCγ1, respectively. The relative pERK1/2, pAKT(S473) and pPLCγ1(Y783) levels for the 5 min time point of PC12-SH2B1β+TrkB cells were used as 1. The error bars represent S.E.M. from three independent experiments. (*: p<0.05, paired Student’s t-test)

Binding of BDNF to TrkB can activate three major intracellular signaling pathways, including MEK-ERK, PI3K-AKT, and PLCγ1 pathways [31]. To determine how SH2B1β promotes BDNF-induced neurite outgrowth, PC12-GFP+TrkB and PC12-SH2B1β+TrkB cell lines were treated with 50 ng/ml BDNF for 5, 10, 30 and 60 min. In PC12-GFP+TrkB cell lines, phosphorylation of ERK1/2 (pERK1/2) was induced by BDNF within 5 min and reduced by 30 min. The level of pERK1/2 remained elevated above the basal level for up to 60 min of BDNF treatment. In contrast, in PC12-SH2B1β+TrkB cell line, pERK1/2 was induced within 5 min after BDNF stimulation and the level of phosphorylation remain higher than that in PC12-GFP+TrkB cells for at least 60 min (Figure 5A). Total ERK1/2 levels serve as the loading control to demonstrate that the difference of pERK1/2 in the two cell lines is not due to the difference in the total amount of ERK1/2. Similarly, phosphorylation of AKT at serine 473 [pAKT(S473)] was induced within 5 min after BDNF treatment in PC12-GFP+TrkB and PC12-SH2B1β+TrkB cells. The level of pAKT(S473) in PC12-SH2B1β+TrkB cells was higher compared to that in PC12-GFP+TrkB cells within 60 min. It is noteworthy that the basal level of pAKT(S473) in PC12-SH2B1β+TrkB cells was higher than that in PC12-GFP+TrkB cells. Moreover, application of BDNF to the two cell lines for 5 min also induced phosphorylation of PLCγ1 at tyrosine 783 [pPLCγ1(Y783)] and the level of pPLCγ1(Y783) was induced up to 60 min. The level of pPLCγ1(Y783) was higher in PC12-SH2B1β+TrkB cells compared to that in PC12-GFP+TrkB cells only for the 5 min time point (Fig. 5B). The quantified results are shown in Figure 5B. These data suggest that overexpression of SH2B1β significantly enhances BDNF-induced pERK1/2 and pAKT(S473).

### Phosphorylation of ERK1/2 and AKT are required for SH2B1β-enhanced neurtie outgrowth

**Figure 6:**
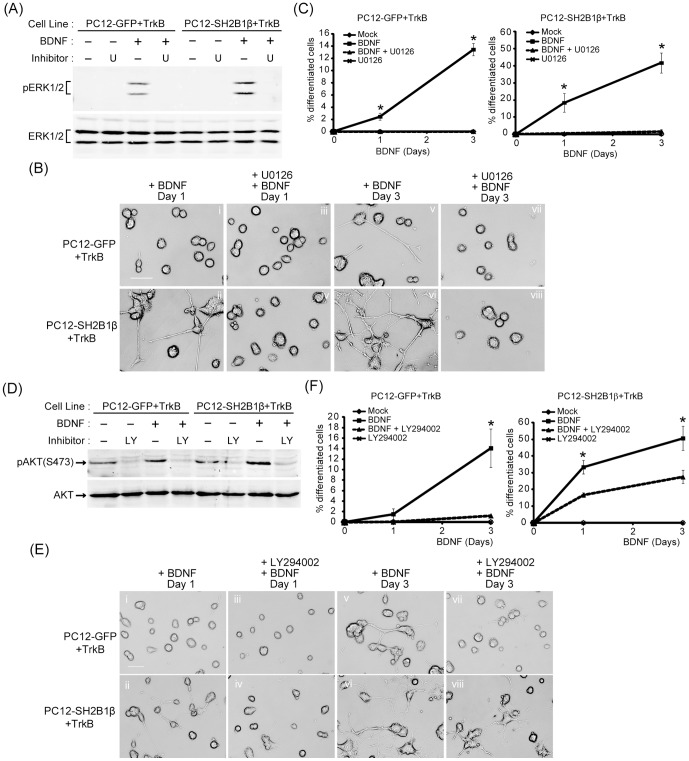
MEK-ERK1/2 and PI3K-AKT signaling pathways are required for SH2B1β-enhanced neurite outgrowth. (A) PC12-GFP+TrkB or PC12-SH2B1β+TrkB cells were incubated in serum-free medium overnight and 20 µM U0126 was added to cells 1 h before 5 min stimulation of 50 ng/ml BDNF. Equal amount of proteins from the cell lysates was resolved with SDS-PAGE and immunoblotted with anti-pERK1/2 antibody. (B) PC12-GFP+TrkB cells (i, iii, v, vii) or PC12-SH2B1β+TrkB cells (ii, iv, vi, viii) were pre-treated with 20 µM U0126 (iii, iv, vii, viii) for 1 h, followed by 50 ng/ml BDNF stimulation in low serum medium. The neurite outgrowth was monitored for 3 days. Representative images of live cells are shown. (C) The percentages of BDNF-induced neuronal differentiation of mock- or U0126-treated PC12-GFP+TrkB and PC12-SH2B1β+TrkB cells were quantified. Values are means ± S.E.M. from three independent experiments. (*: p<0.05, paired Student’s t-test) (D) PC12-GFP+TrkB or PC12-SH2B1β+TrkB cells were incubated in serum-free medium overnight and 20 µM LY294002 was added to cells 1 h before 5 min stimulation of 50 ng/ml BDNF. Equal amount of cell lysates was resolved with SDS-PAGE and immunoblotted with anti-pAKT(S473) antibody. (E) PC12-GFP+TrkB cells (i, iii, v, vii) or PC12-SH2B1β+TrkB cells (ii, iv, vi, viii) were pre-treated with 20 µM LY294002 (iii, iv, vii, viii) for 1 h, followed by 50 ng/ml BDNF stimulation in low serum medium. The neurite outgrowth was monitored for 3 days. Representative images of live cells are shown. (F) The percentages of BDNF-induced neuronal differentiation of mock- or LY294002-treated PC12-GFP+TrkB and PC12-SH2B1β+TrkB cells were quantified. Values are means ± S.E.M. from three independent experiments. (*: p<0.05, paired Student’s t-test)

To determine whether MEK-ERK1/2 and PI3K-AKT signaling pathways are required for BDNF-induced neurite outgrowth, specific inhibitors were used to block either one of these pathways. PC12-GFP+TrkB and PC12-SH2B1β+TrkB cells were stimulated by 50 ng/ml BDNF for the indicated time in the presence or absence of selective pharmacological inhibitors of the MAPK or PI3K-AKT pathways and the neurite outgrowth was analyzed. The MEK inhibitor, U0126, inhibited BDNF-induced pERK1/2 in both cell lines (Figure 6A). The BDNF-induced neurite outgrowth of PC12-GFP+TrkB cells was completely abolished by the U0126 (Figure 6B, panel v and vii). Similar to the result for PC12-GFP+TrkB cells, neurite outgrowth was blocked by U0126 treatment for PC12-SH2B1β+TrkB cells (Figure 6B, panel ii, iv, vi and viii). Percentages of neuronal differentiation of PC12-GFP+TrkB and PC12-SH2B1β+TrkB cells were significantly reduced in the presence of U0126 (Figure 6C). Similarly, PI3K inhibitor, LY294002, inhibited BDNF-induced pAKT(S473) in both cell lines (Figure 6D). Inhibition of the PI3K-AKT pathway blocked BDNF-induced neurite outgrowth of PC12-GFP+TrkB (Figure 6E, panel i, iii, v and vii). In contrast, neurite outgrowth was reduced but not completely inhibited by LY294002 for PC12-SH2B1β+TrkB cells (Figure 6E, panel ii, iv, vi and viii). As a result, the percentages of differentiation of PC12-GFP+TrkB and PC12-SH2B1β+TrkB cells were reduced by inhibiting PI3K-AKT (Figure 6F). Taken together, these results suggest that MEK-ERK1/2 and PI3K-AKT signaling pathways are required for BDNF-induced neurite outgrowth promoted by SH2B1β.

### SH2B1β enhances BDNF-induced phosphorylation of STAT3 at serine 727 through MEK-ERK1/2 and PI3K-AKT signaling pathways

**Figure 7:**
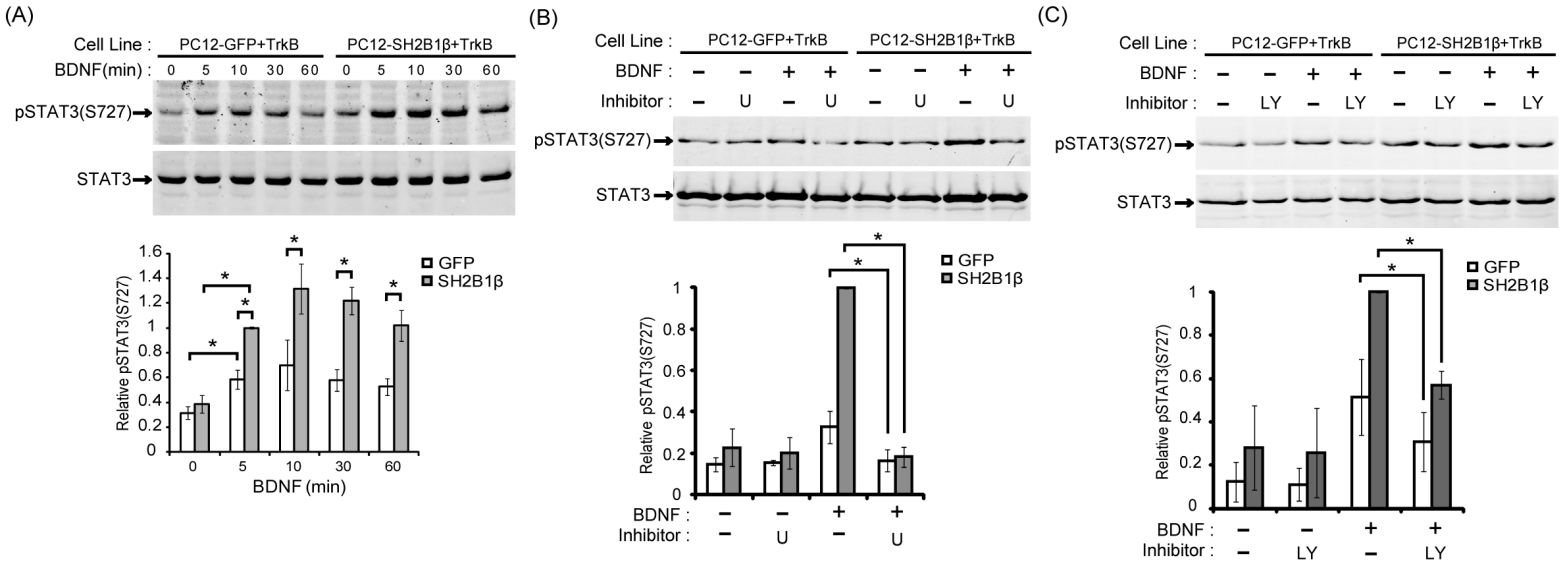
SH2B1β enhances BDNF-induced pSTAT3(S727) through MEK-ERK1/2 and PI3K-AKT signaling pathways. (A) PC12-GFP+TrkB or PC12-SH2B1β+TrkB cells were incubated in serum-free medium overnight before stimulation with 50 ng/ml BDNF for the indicated time points. Lysates were collected and equal amount of proteins was separated by SDS-PAGE and immunoblotted with either anti-pSTAT3(S727) or anti-STAT3 antibody. Representative blots from three independent experiments are shown. pSTAT3(S727) level was normalized to total STAT3 in PC12-GFP+TrkB and PC12-SH2B1β+TrkB cells both treated with BDNF and the relative pSTAT3(S727) level for the 5 min time point of PC12-SH2B1β+TrkB cells was used as 1. The error bars represent S.E.M. from three independent experiments. (*: p<0.05, paired Student’s t-test) (B) Cells were incubated in serum-free medium for 16 h, then U0126 (20 µM) was added to cells 1 h before 50 ng/ml BDNF stimulation for 5 min. Cell lysate was collected and equal amount of proteins was analyzed with SDS-PAGE and immunoblotted with anti-pSTAT3(S727) antibody. pSTAT3(S727) level was normalized to total STAT3 in PC12-GFP+TrkB and PC12-SH2B1β+TrkB cells both treated with either BDNF or U0126 and the relative pSTAT3(S727) level for the 5 min time point of BDNF stimulation in PC12-SH2B1β+TrkB cells was used as 1. The error bars represent S.E.M. from free independent experiments. (*: P<0.05, paired Student’s t-test) (C) PC12-GFP+TrkB or PC12-SH2B1β+TrkB cells were treated with 20 µM LY294002 for the indicated time points and processed as in (B). Lysates were collected and equal amount of proteins was separated by SDS-PAGE and immunoblotted with either anti-pSTAT3(S727) or anti-STAT3 antibody. Representative blots from three independent experiments are shown. The relative pSTAT3(S727) level was normalized as described in (B). The error bars represent S.E.M. from three independent experiments. (*: P<0.05, paired Student’s t-test).

BDNF has been reported to induce phosphorylation of signal transducer and activator of transcription 3 (STAT3) at serine 727 [pSTAT3 (S727)] in cortical neurons [32]. This result prompted us to investigate whether BDNF also induces pSTAT3(S727) in PC12-TrkB cells. To this end, PC12-GFP+TrkB and PC12-SH2B1β+TrkB cells were treated with 50 ng/ml BDNF for the 5, 10, 30 and 60 min. BDNF induced pSTAT3(S727) within 5 min and the level of phosphorylation remained elevated above the basal level for up to 60 min for both cell lines. The relative pSTAT3(S727) in PC12-SH2B1β+TrkB cells was higher compared to that in PC12-GFP+TrkB cells at the indicated time points (Figure 7A). This result suggests that overexpressing SH2B1β enhances BDNF-induced pSTAT3(S727). To investigate which pathway is responsible for the induced pSTAT3(S727), specific inhibitors of MEK-ERK1/2 and PI3K-AKT pathways were used to examine the contribution of these two pathways to BDNF-induced pSTAT3(S727). Inhibition of MEK-ERK1/2 pathway by U0126 reduced BDNF-induced pSTAT3(S727) in both PC12-GFP+TrkB and PC12-SH2B1β+TrkB cells (Figure 7B). Furthermore, the PI3K inhibitor, LY294002, also reduced pSTAT3(S727) in both cell lines (Figure 7C). These data demonstrate that both the activation of MEK-ERK1/2 and PI3K-AKT signaling pathways contribute to BDNF-induced pSTAT3(S727).

### SH2 domain of SH2B1β is required for SH2B1β-mediated enhancement of BDNF-induced neurite outgrowth

**Figure 8:**
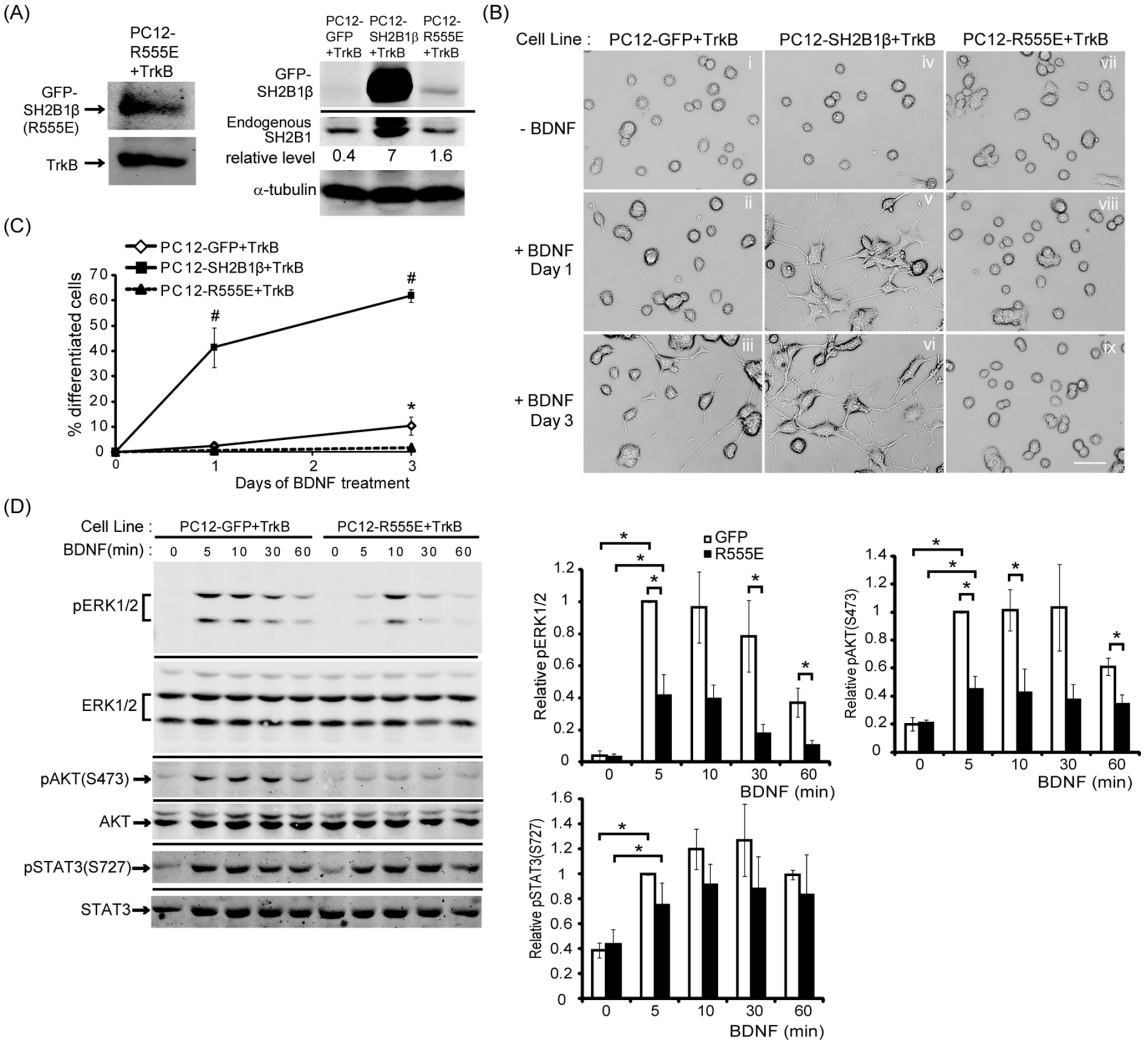
SH2 domain of SH2B1β is required to enhance BDNF-induced neurite outgrowth and signaling. PC12 cells overexpressing GFP and TrkB (PC12-GFP+TrkB), GFP-SH2B1β and TrkB (PC12-SH2B1β+TrkB) or GFP-SH2B1β(R555E) and TrkB (PC12-R555E+TrkB) were established. (A) Cell lysates from PC12-GFP+TrkB, GFP-SH2B1β and PC12-R555E+TrkB cells were analyzed via SDS-PAGE and immunoblotted with anti-SH2B1 or TrkB antibody to determine the expression of GFP-SH2B1β, GFP-SH2B1β(R555E), endogenous SH2B1 and TrkB. The relative expression levels of GFP-SH2B1β and GFP-SH2B1β(R555E) were normalized to the respective endogenous SH2B1 level in each cell line. (B) PC12-GFP+TrkB cells (i, iii, v), PC12-SH2B1β+TrkB cells (ii, iv, vi) or PC12-R555E+TrkB cells (vii, viii, ix) were treated with 100 ng/ml BDNF in low serum medium for 0 (i, iv, vii), 1 (ii, v, viii) or 3 (iii, vi, ix) days. Representative images of live cells are shown. Scale car  =  50 µm. (C) The percentage of differentiated cells for PC12-GFP+TrkB, PC12-SH2B1β+TrkB or PC12-R555E+TrkB cells treated with BDNF for 1 or 3 days was counted. Values are means ± S.E.M. from three independent experiments (*, **#**: p<0.05, paired Student’s t-test. *: comparison between PC12-GFP+TrkB and PC12-R555E+TrKB cells for the same BDNF treatment day; **#**: comparison between PC12-SH2B1β+TrkB and PC12-R555E+TrKB cells for the same BDNF treatment day). (D) PC12-GFP+TrkB or PC12-R555E+TrkB cells were incubated in serum-free medium overnight before stimulation with 50 ng/ml BDNF for the indicated time points. Lysates were collected and equal amount of proteins was separated by SDS-PAGE and immunoblotted with anti-pERK1/2, ERK1/2, pAKT(S473), AKT, pSTAT3(S727) and STAT3 antibodies. Representative blots from three independent experiments are shown. pERK1/2, pAKT(S473), and pSTAT3(S727) level were normalized to total ERK1/2, AKT, and STAT3, respectively and the relative pERK1/2, pAKT(S473), and pSTAT3(S727) levels for the 5 min time point of PC12-GFP+TrkB cells were used as 1. The error bars represent S.E.M. from three independent experiments. (*: p<0.05, paired Student’s t-test).

SH2 domain is known to interact with receptor tyrosine kinases. To determine whether SH2 domain of SH2B1β is responsible for BDNF-induced neurite outgrowth, we established a PC12 stable cell line that stably expresses SH2B1β(R555E), a dominant negative mutant with a point mutation at the FLVR motif within the SH2 domain and TrkB receptors (PC12-R555E+TrkB). The expressions of GFP-SH2B1β, GFP-SH2B1β(R555E), endogenous SH2B1 and TrkB are shown in Figure 8A. To examine the effects of SH2B1β(R555E) on BDNF-induced neurite outgrowth, PC12-GFP+TrkB, PC12-SH2B1β+TrkB and PC12-R555E+TrkB cell lines were treated with 100 ng/ml BDNF for 1 or 3 days. The relative neurite outgrowth of PC12-R555E+TrkB cells was significantly less than those of PC12-GFP+TrkB and PC12-SH2B1β+TrkB cells (Figure 8B). The percentages of BDNF-induced neuronal differentiation of PC12-R555E+TrkB cells were also the lowest compared to PC12-GFP+TrkB and PC12-SH2B1β+TrkB cells (Figure 8C). These results suggest that SH2 domain of SH2B1β is required to promote BDNF-induced neurite outgrowth.

To examine how SH2B1β(R555E) may negatively regulate BDNF-induced neurite outgrowth, BDNF-induced signaling pathways between PC12-GFP+TrkB and PC12-R555E+TrkB cells were compared. PC12-GFP+TrkB and PC12-R555E+TrkB cells were treated with 50 ng/ml BDNF for 5, 10, 30 and 60 min. As demonstrated in Figure 8D, BDNF-induced phosphorylation of ERK1/2 and AKT in PC12-R555E+TrkB cells was reduced compared to that in PC12-GFP cells. These results strongly suggest that SH2 domain of SH2B1β is required for enhancing BDNF-induced signaling.

### Tyrosine phosphorylation of SH2B1β is involved in BDNF-induced neurite outgrowth and signaling

**Figure 9:**
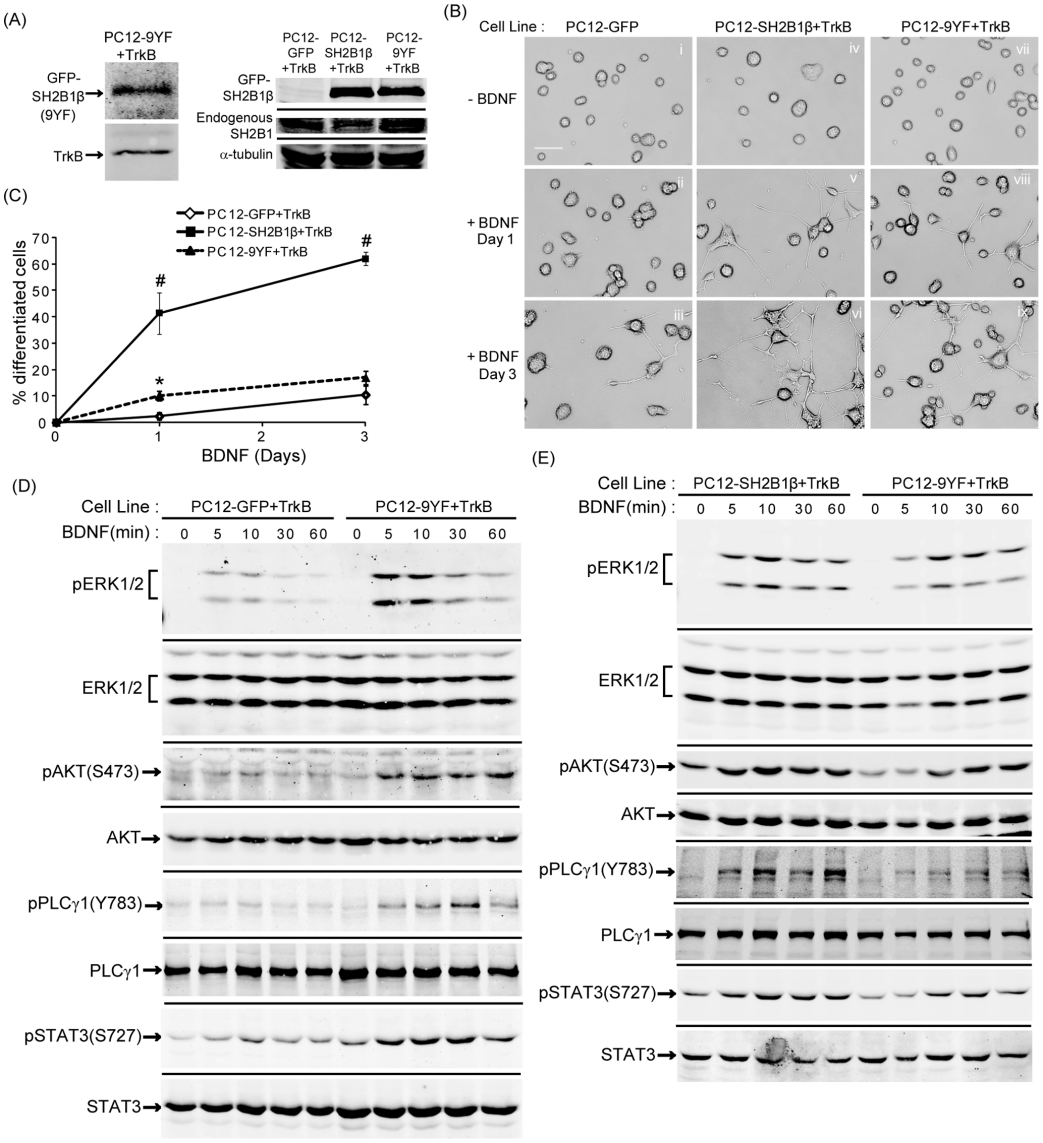
The tyrosine phosphorylation of SH2B1β contributes to SH2B1β-mediated enhancement of BDNF-induced neurite outgrowth and signaling. (A) Cell lysates from PC12-GFP+TrkB, PC12-SH2B1β+TrkB and PC12-9YF+TrkB cells were analyzed via SDS-PAGE and immunoblotted with anti-SH2B1 or TrkB antibody to determine the expression of GFP-SH2B1β, GFP-SH2B1β(9YF), endogenous SH2B1 and TrkB. (B) PC12-GFP+TrkB cells (i, ii, iii), PC12-SH2B1β+TrkB cells (iv, v, vi) or PC12-9YF+TrkB cells (vii, viii, ix) were treated with 100 ng/ml BDNF in low serum medium for 0 (i, iv, vii), 1 (ii, v, viii) or 3 (iii, vi, ix) days. Representative live cell images are shown. (C) The percentage of differentiated cells in PC12-GFP+TrkB, PC12-SH2B1β+TrkB cells or PC12-9YF+TrkB was counted after BDNF treatment for 1 or 3 days. Values were means ± S.E.M from three independent experiments. (*: compared to the percentage of PC12-9YF+TrkB cells for the same day of BDNF treatment, p<0.05, paired Student’s t-test; #: compared to the percentage of PC12-GFP+TrkB cells for the same BDNF treatment day, p<0.05, paired Student’s t-test) (D) PC12-GFP+TrkB or PC12-9YF+TrkB cells were incubated in serum-free medium overnight before stimulation with 50 ng/ml BDNF for the indicated time points. Lysates were collected and equal amount of proteins was separated by SDS-PAGE and immunoblotted with anti-pERK1/2, ERK1/2, pAKT(S473), AKT, pPLCγ1(Y783), PLCγ1, pSTAT3(S727), and STAT3 antibodies. Representative blots from two independent experiments are shown. (E) PC12-SH2B1β+TrkB or PC12-9YF+TrkB cells were incubated in serum-free medium overnight before stimulation with 50 ng/ml BDNF for the indicated time points. Lysates were collected and equal amount of proteins was separated by SDS-PAGE and immunoblotted with anti-pERK1/2, ERK1/2, pAKT(S473), AKT, pPLCγ1(Y783), PLCγ1, pSTAT3(S727) and STAT3 antibodies. Representative blots from two independent experiments are shown.

SH2B1 is reported to be phosphorylated by several of its receptor tyrosine kinase binding partners, including the receptors for insulin, PDGF, NGF, and FGF3 [21], [22], [24], [25], [33]. SH2B1β contains nine tyrosines. To examine whether phosphorylation of the nine tyrosines within SH2B1β regulates BDNF-induced neurite outgrowth, all nine tyrosines of SH2B1β were mutated to phenylalanine to generate SH2B1β(9YF). PC12 stable cell lines expressing SH2B1β(9YF) and TrkB (PC12-9YF+TrkB) were established. Overexpression of SH2B1β(9YF), TrkB and endogenous SH2B1 in PC12-9YF+TrkB are shown in Figure 9A. The relative overexpression levels of SH2B1β and SH2B1β(9YF) were similar. BDNF-induced neurite outgrowth and neuronal differentiation were slightly better than control PC12-GFP+TrkB cells but significantly worse compared to PC12-SH2B1β+TrkB cells (Figure 9B-C). BDNF-induced pERK1/2, pAKT, pPLCγ and pSTAT3(S727) in PC12-9YF+TrkB cells were higher than control cells (Figure 9D) but lower than PC12-SH2B1β+TrkB cells (Figure 9E). The quantified results are shown in Figure S1. These results indicate that tyrosine phosphorylation of SH2B1β contributes to SH2B1β-mediated enhancement of BDNF-induced neurite outgrowth. Nonetheless, these results also suggest that tyrosine phosphorylation is not the only mechanism that SH2B1β enhances neurite outgrowth.

### SH2B1β interacts with TrkB receptors

**Figure 10:**
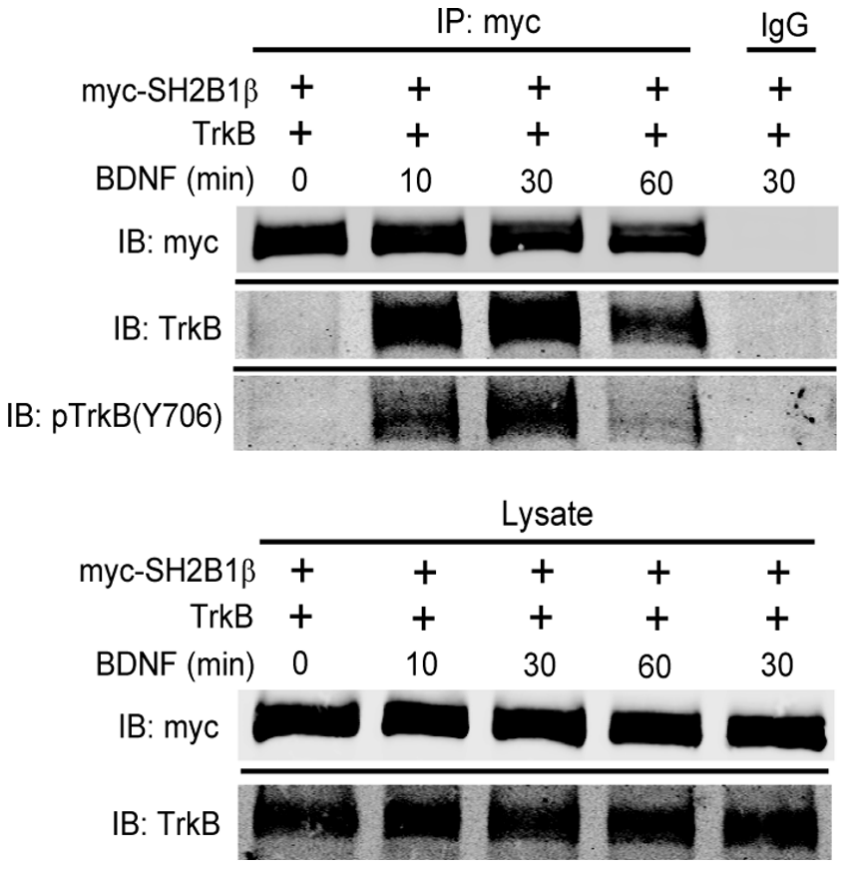
SH2B1β interacts with TrkB. Lysates from 293T cells co-transfected with myc-SH2B1β and TrkB followed by 50 ng/ml BDNF stimulation for 0, 10, 30, 60 minutes were collected and subjected to immunoprecipitation using anti-myc antibody followed by immunoblotting with anti-myc, TrkB or pTrkB(Y706) antibody. IgG was used as negative control. Representative blots are shown from two independent experiments.

A central question to address is how SH2B1β may enhance BDNF signaling. One possibility is that SH2B1β interacts with TrkB. To this end, 293T cells were co-transfected with TrkB and myc-SH2B1β followed by BDNF treatment. SH2B1β was immunoprecipitated using anti-myc antibody. In response to BDNF for 10 or 30 min, the association between SH2B1β, TrkB and pTrkB(Y706) was increased (Figure 10, upper panels). Phosphorylation of TrkB at tyrosine 706 was induced in response to BDNF treatment for 10 and 30 min (Figure 10, lower panels). This result suggests that BDNF-induced phosphorylation and activation of TrkB facilitates its interaction with SH2B1β. This interaction may be responsible for the enhanced and prolonged BDNF signaling by overexpression of SH2B1β.

## Discussion

The main purpose of this study is to investigate how SH2B1 regulates BDNF-induced signaling and thus morphogenesis of cortical neurons and PC12-TrkB cells. SH2B1β enhances BDNF-induced phosphorylation of ERK1/2, AKT and STAT3. Inhibition of MEK-ERK1/2 pathway clearly blocks BDNF-induced neurite outgrowth for both PC12-SH2B1β+TrkB and PC12-GFP+TrkB cells. Inhibiting PI3K-AKT pathway, on the other hand, blocks neurite outgrowth of PC12-GFP+TrkB cells but only reduces neurite outgrowth of PC12-SH2B1β+TrkB cells. These results suggest that SH2B1β enhances BDNF-induced neurite outgrowth mainly through MEK-ERK1/2 pathway. In line with this conclusion, the basal level of pAKT(S473) in PC12-SH2B1β+TrkB cells was higher than that of control cells whereas no spontaneous neurite outgrowth was found. Because pAKT was shown to be the major survival-promoting factor for neurons [34], it is possible that the enhanced pAKT in PC12-SH2B1β+TrkB cells serve to promote cell survival. Our recent report showing that overexpression of SH2B1β reduces oxidative stress-induced cell death supports this possibility [35]. Alternatively, SH2B1β may enhance pAKT to increase dendritic formation and/or branches of cortical and hippocampal neurons.

SH2 domain of SH2B1 is responsible for binding to a variety of its receptor tyrosine kinases, including the receptors for insulin, PDGF, NGF and GDNF. In this study, we demonstrate that the binding of SH2B1β to TrkB. The dominant negative mutant of SH2B1β that lacks functional SH2 domain cannot enhance BDNF signaling and neurite outgrowth. SH2B1 has been reported to be phosphorylated in response to activation of PDGFR, TrkA and FGFR3 [21], [22], [24], [25], [33]. SH2B1β(9YF), with all nine tyrosines mutated, is able to slightly enhance BDNF-induced neurite outgrowth and signaling. These results raise a possibility that, in addition to mechanisms regulated by tyrosine phosphorylation of SH2B1, other SH2B1-regulated functions may play a role during BDNF-induced neuronal differentiation.

Both BDNF and SH2B1 have been implicated in metabolic diseases. SH2B1 knockout mice develop obesity, diabetes and insulin resistance [36]–[38]. Human subjects with SH2B1 mutations have also been reported to result in hyperphagia, obesity, insulin resistance, reduced height and behavioral abnormalities [39]–[42]. While peripheral insulin resistance attributes to the pathogenesis of diabetes, brain insulin resistance has been associated with Alzheimer’s disease (AD). Insulin signaling and regulation are critical for neuronal survival, metabolism and energy generation necessary for memory and cognition [43], [44]. Previous studies from our group and others showed that SH2B1 promotes neuronal differentiation and reduces oxidative stress-induced cell death [24], [29], [35], [38]. SH2B1 also regulates insulin signaling [33], [45]. Thus, it is likely that SH2B1 may have roles not only in neuronal development and survival, but also metabolism. BDNF has also been shown to correlate with metabolic syndrome as well as diabetes [46]. Plasma level of BDNF in diabetic patients is lower than normal individuals [47] and administrating BDNF to transgenetic diabetic animals normalizes blood glucose [48]. Although the exact mechanisms by which SH2B1 and BDNF regulate metabolism are not clear, our study shows an association between SH2B1 and TrkB, suggesting a link of SH2B1 in regulating BDNF signaling and glucose metabolism.

Taken together, this study suggests that the adaptor protein SH2B1β promotes BDNF-induced neurite outgrowth in part through enhancing BDNF-induced phosphorylation of ERK1/2 and AKT. SH2B1β does so through binding to TrkB. In response to BDNF, tyrosine phosphorylation of SH2B1β is likely to play a role in enhancing BDNF-induced signaling and neurite outgrowth.

## Supporting Information

Figure S1 **The tyrosine phosphorylation of SH2B1β contributes to SH2B1β-mediated enhancement of BDNF-induced signaling.** PC12-GFP+TrkB, PC12-SH2B1β+TrkB and PC12-SH2B1β(9YF)+TrkB cells were cultured in serum-free medium overnight before stimulation of 50 ng/ml BDNF for the indicated time points. Lysates were collected and equal amount of proteins was separated by SDS-PAGE and immunoblotted with anti-pERK1/2, ERK1/2, pAKT(S473), AKT, pSTAT3(S727), and STAT3 antibodies. (A) pERK1/2, pAKT(S473), and pSTAT3(S727) levels were normalized to total ERK1/2, AKT and PLCγ1, respectively. The relative pERK1/2, pAKT(S473) and pSTAT3(S727) levels for the 5 min time point of PC12-SH2B1β(9YF)+TrkB cells were used as 1. The error bars represent S.D., indicating the range of data from two independent experiments. (B) The relative pERK1/2, pAKT(S473), and pSTAT3(S727) levels were normalized as described in (B). The relative pERK1/2, pAKT(S473) and pSTAT3(S727) levels for the 5 min time point of PC12-SH2B1β+TrkB cells were used as 1. The error bars represent S.D. indicating the range of data from two independent experiments.
(TIF)

Figure S2 **SH2B1β regulates BDNF-induced neurite outgrowth and branching in hippocampal and cortical neurons.** (A) E18 primary hippocampal neurons were transiently co-transfected with GFP and shLacZ or shSH2B1 on DIV 4. One day after transfection, neurons were treated with 50 ng/ml BDNF for 2 days. (B) E18 primary cortical neurons were transiently transfected with either GFP or GFP-SH2B1β on DIV 4. One day after transfection, neurons were treated with 50 ng/ml BDNF for 2 days. The morphology of the neurons was visualized on DIV 7 by Zeiss LSM510 meta confocal microscope using 20X (NA/0.75) or 40X (NA/0.75) objectives. Boxes mark the neurites of hippocampal or cortical neurons. Enlarged images of the neurites and branching are shown on the right panels. Scale bar  =  20 µm.
(TIF)
