# Supplementary figures and images for: New Function of the Adaptor Protein SH2B1 in Brain-Derived Neurotrophic Factor-Induced Neurite Outgrowth
Source: PLoS One. 2013 Nov 15;8(11):e79619. doi: 10.1371/journal.pone.0079619 (PMC3829828; doi:10.1371/journal.pone.0079619)

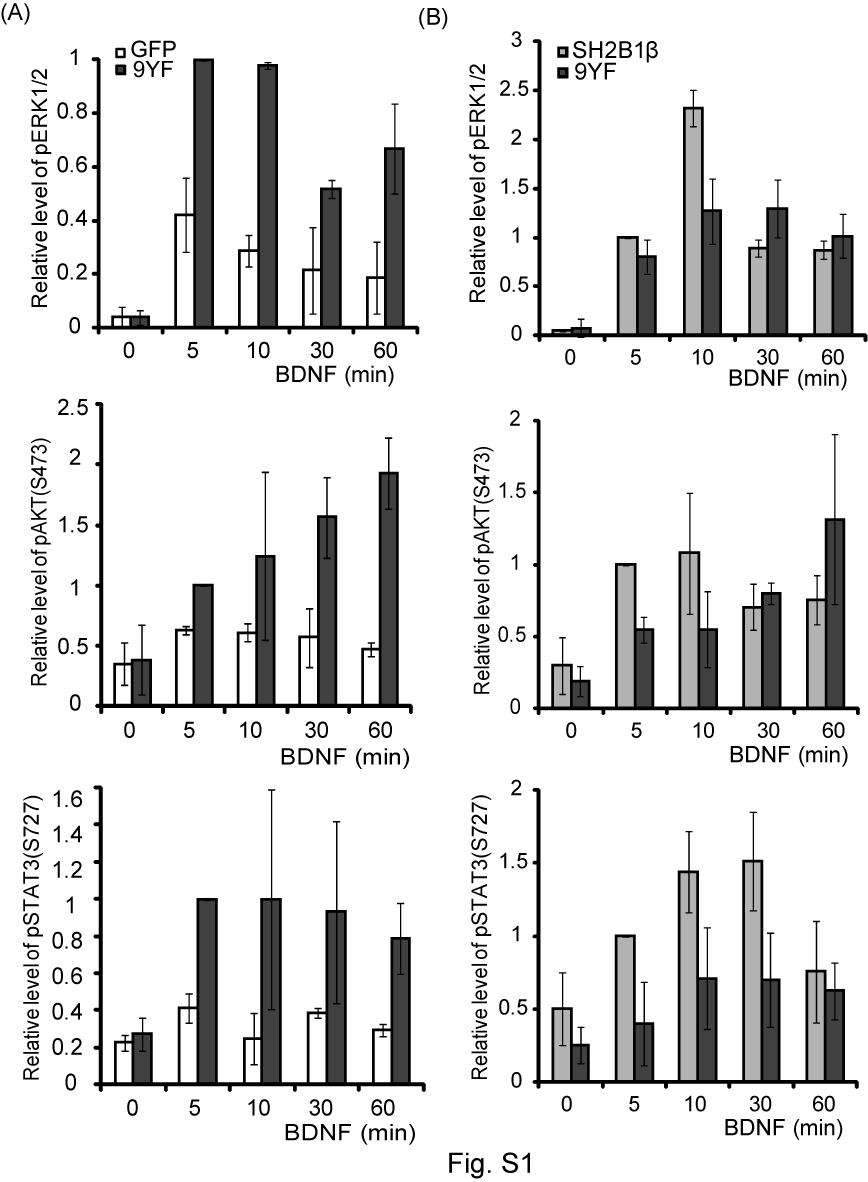

Supplement: Figure S1 — The tyrosine phosphorylation of SH2B1β contributes to SH2B1β-mediated enhancement of BDNF-induced signaling. PC12-GFP+TrkB, PC12-SH2B1β+TrkB and PC12-SH2B1β(9YF)+TrkB cells were cultured in serum-free medium overnight before stimulation of 50 ng/ml BDNF for the indicated time points. Lysates were collected and equal amount of proteins was separated by SDS-PAGE and immunoblotted with anti-pERK1/2, ERK1/2, pAKT(S473), AKT, pSTAT3(S727), and STAT3 antibodies. (A) pERK1/2, pAKT(S473), and pSTAT3(S727) levels were normalized to total ERK1/2, AKT and PLCγ1, respectively. The relative pERK1/2, pAKT(S473) and pSTAT3(S727) levels for the 5 min time point of PC12-SH2B1β(9YF)+TrkB cells were used as 1. The error bars represent S.D., indicating the range of data from two independent experiments. (B) The relative pERK1/2, pAKT(S473), and pSTAT3(S727) levels were normalized as described in (B). The relative pERK1/2, pAKT(S473) and pSTAT3(S727) levels for the 5 min time point of PC12-SH2B1β+TrkB cells were used as 1. The error bars represent S.D. indicating the range of data from two independent experiments. (TIF) [file pone.0079619.s001.tif]

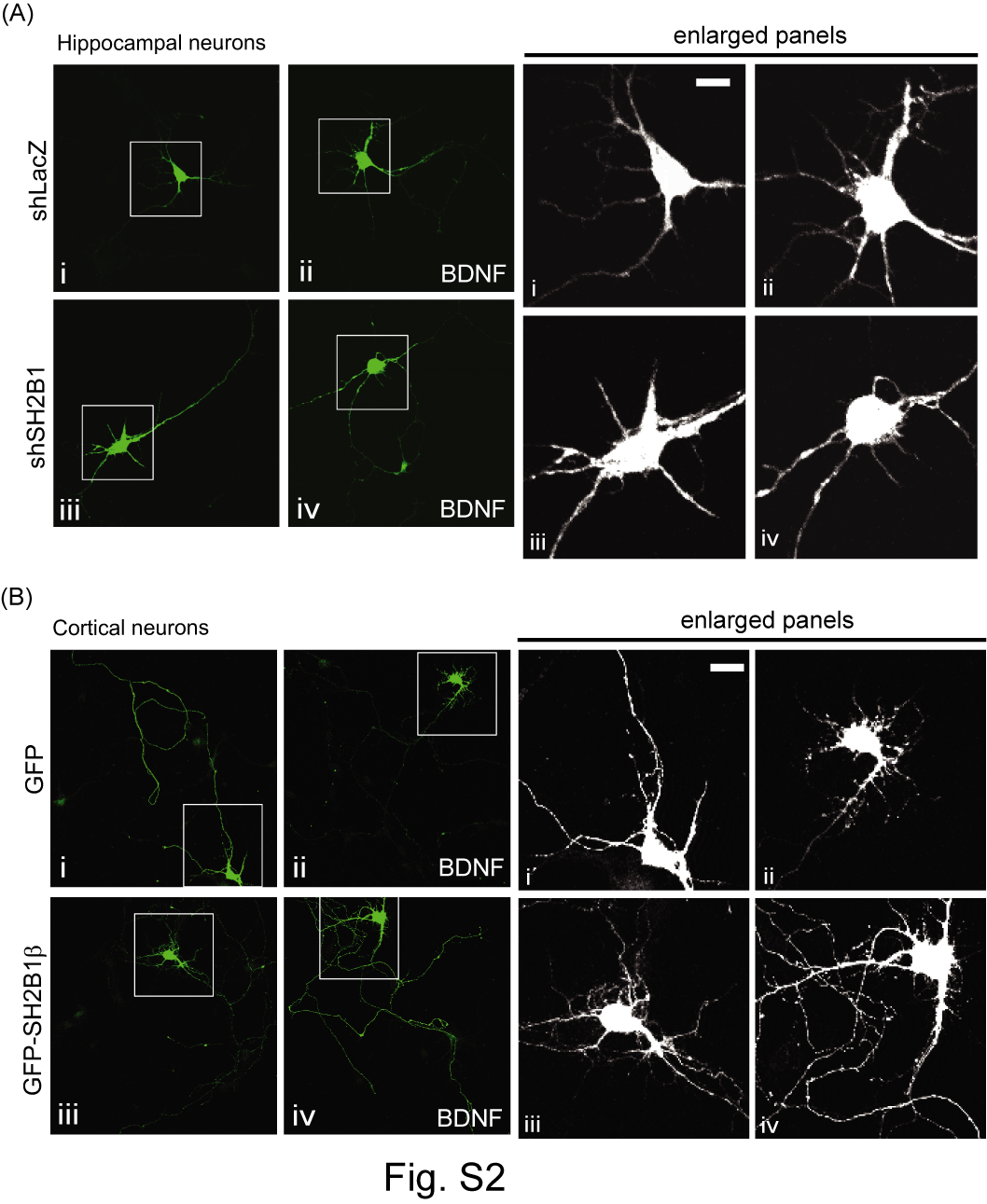

Supplement: Figure S2 — SH2B1β regulates BDNF-induced neurite outgrowth and branching in hippocampal and cortical neurons. (A) E18 primary hippocampal neurons were transiently co-transfected with GFP and shLacZ or shSH2B1 on DIV 4. One day after transfection, neurons were treated with 50 ng/ml BDNF for 2 days. (B) E18 primary cortical neurons were transiently transfected with either GFP or GFP-SH2B1β on DIV 4. One day after transfection, neurons were treated with 50 ng/ml BDNF for 2 days. The morphology of the neurons was visualized on DIV 7 by Zeiss LSM510 meta confocal microscope using 20X (NA/0.75) or 40X (NA/0.75) objectives. Boxes mark the neurites of hippocampal or cortical neurons. Enlarged images of the neurites and branching are shown on the right panels. Scale bar = 20 µm. (TIF) [file pone.0079619.s002.tif]
